# An unusual presentation of childhood lichen planus successfully treated with 308‐nm excimer laser

**DOI:** 10.1111/jocd.16522

**Published:** 2024-08-09

**Authors:** Dorsaf Mzoughi, Faten Rabhi, Malek Ben Slimane, Ines Mallek, Kahena Jaber, Mohamed Raouf Dhaoui

**Affiliations:** ^1^ Dermatology Department Military Hospital of Tunis Tunis Tunisia; ^2^ Faculty of medicine of Tunis Tunis El Manar University Tunis Tunisia; ^3^ Histopathology Department Military Hospital of Tunis Tunis Tunisia

To the Editor,

A 4‐year‐old infant was referred to our department for assessment of a skin lesion on the left elbow that appeared 9 months ago. Physical examination showed multiple firm erythematous papules with fine scaling on the left elbow (Figure 1A). No other abnormalities of the skin, the mucous membranes, hair, or nails were noted. No history of itching was reported by the patient. Dermoscopy showed an erythematous background with linear vessels in a radial pattern, yellow spots in the center and Wickham striae (Figure 2A). A skin biopsy was performed. Histopathologic examination showed orthokeratotic hyperkeratosis, hypergranulosis, acanthosis, and hyaline bodies in the deep layers of the epidermis, as well as a lymphocytic dense band‐like infiltration of the dermis (Figure 2B) thus concluding in a lichen planus (LP). The patient was treated initially with high potency topical corticosteroids (betamethasone dipropionate) once a day regularly for 6 weeks without improvement. Then, he underwent 308‐nm Excimer laser (EL) therapy. We started the treatment at 100 mJ/cm^2^ at first session then we increased in steps of 50 mJ/cm^2^ per session (two sessions per week: at a rate of a session every 4 days). A protective eyewear was provided during the treatment. Within 10 sessions, a 100% clearance of the cutaneous lesions was achieved with no relapse for 2 years (Figure 1B). The patient did not show any adverse events during or after the EL therapy.

**FIGURE 1:**
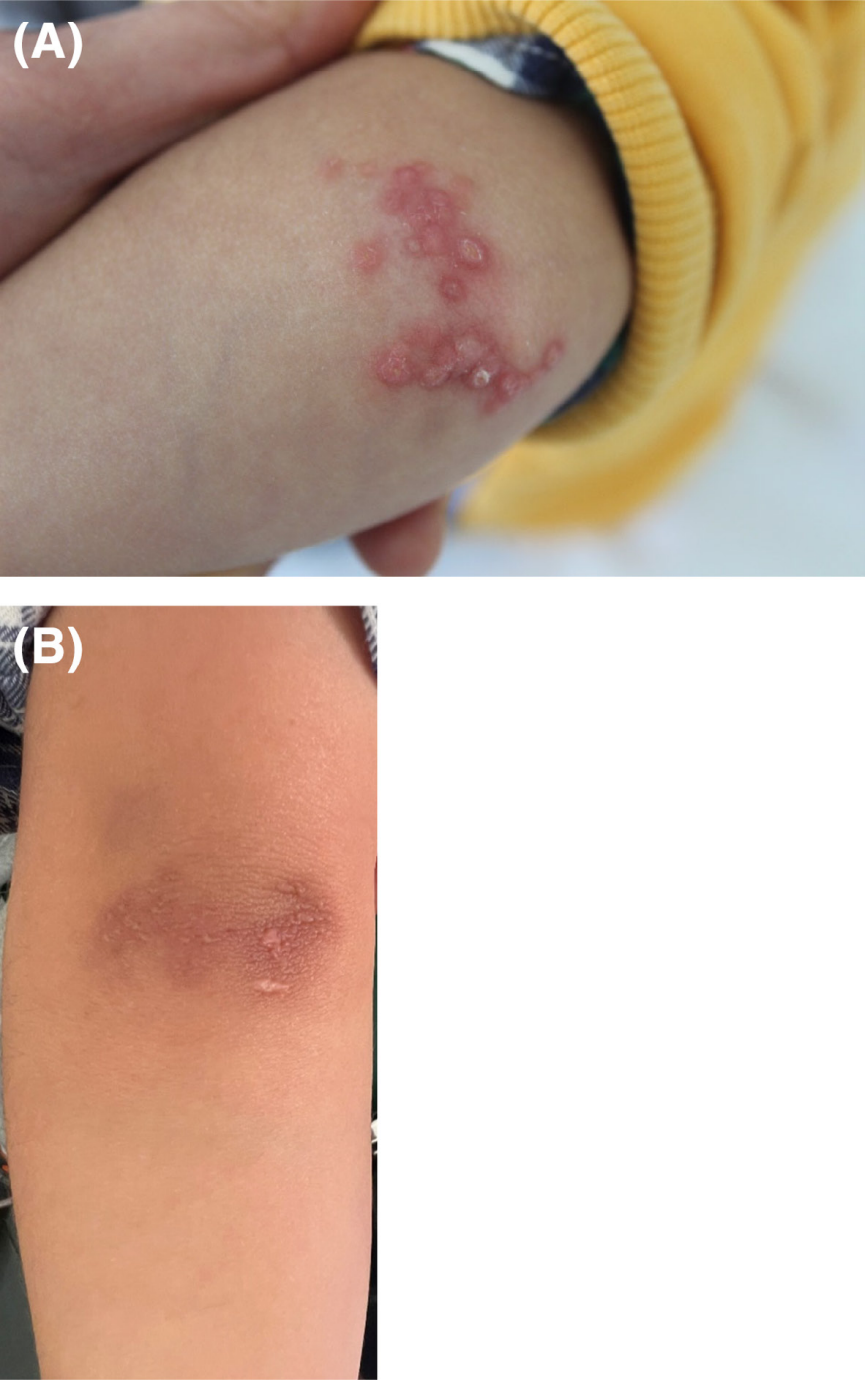
(A) Multiple firm erythematous papules with fine scaling on the left elbow, (B) Clearance of LP lesions after excimer laser treatment.

**FIGURE 2:**
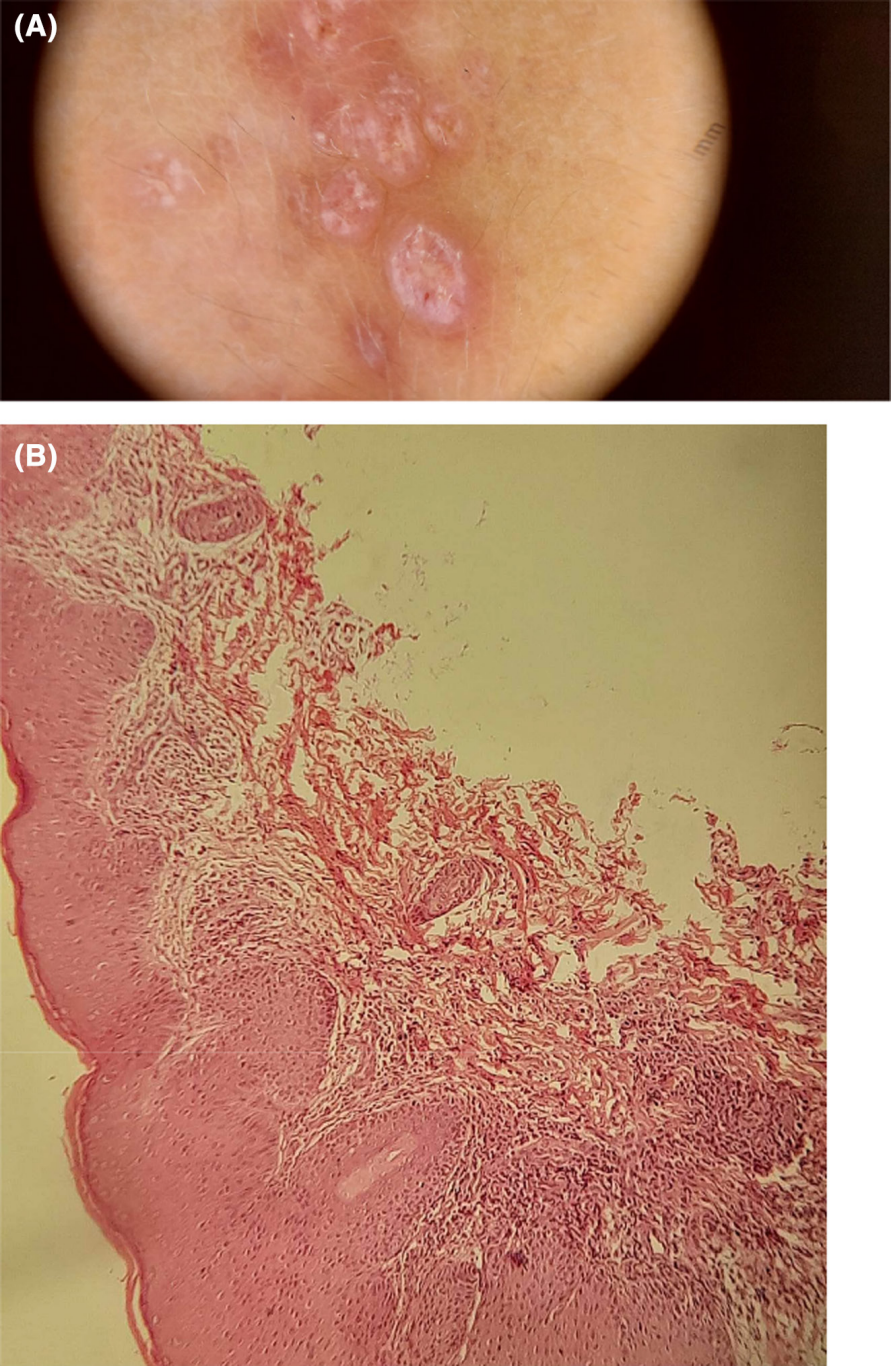
(A) Dermoscopy showing an erythematous background with linear vessels in a radial pattern, yellow spots in the center and Wickham striae, (B) Biopsy specimen demonstrating orthokeratotic hyperkeratosis, hypergranulosis, acanthosis, and a lymphocytic dense band‐like infiltrate (HE*20).

Lichen planus (LP) is a common inflammatory skin condition. The prevalence of the disease is 1% in the general population.^1^ It is typically characterized by itchy, violaceous, polygonal, symmetrical, and bilateral papules frequently covered by a lacy network of white scales on their surface, known as Wickham striae.^2^ LP more commonly affects adults between 30 and 60 years and is rare in children occurring in 2%–3% of total LP cases.^3^ Although classical LP is observed in most of cases, childhood LP (CHLP) may present specific clinical features including linear, annular, bullous, hypertrophic, and follicular variants. Mucosa, scalp, and nail involvement (nail dystrophy, atrophy, ridging, and pterygium formation) are not usually associated with skin LP in children.^4^

The most common site of onset in CHLP is the limbs, more commonly the lower limbs. Our patient had non itchy unilateral lesions. Reported precipitating factors include upper respiratory tract infection and viral exanthema.^2^ Some children developed a lichenoid eruption after antihepatitic B vaccination.^4^

Differential diagnoses of CHLP vary according to morphology and site of involvement.^2^ The main ones to be mentioned especially in our case are frictional lichenoid dermatitis, plane warts, lichen simplex chronicus, lichen amyloidosis, and lichenoid psoriasis.^2^

There is no consensus regarding the treatment of CHLP. Topical corticosteroids and oral antihistamines remain the treatment of choice in most patients with localized classic disease. Intralesional triamcinolone may be used for both oral and cutaneous LP if the child can be convinced about the procedure. Topical tacrolimus has been also used successfully in CHLP.^2^ For extensive or resistant LP, dapsone, oral corticosteroids (1–2 mg/kg/day for a 1–2‐week period), and oral acitretin (0.5 mg/kg/day) have been used.^4^ Other options for cutaneous LP are antimalarials, thalidomide, cyclosporine, azathioprine, and mycophenolate mofetil.^2^ On the contrary, Psoralen plus Ultraviolet A (PUVA) therapy is not indicated in children but Ultraviolet B (UVB) phototherapy has been tried and proved to be safe and effective in children with acute widespread LP.^2^, ^4^ Pavlotsky and et al. reported a satisfying response in 70% of the cases, and 85% of those were still in remission after a median of 34.7 months.^2^ The complete response rate and the need for higher cumulative exposure doses were not influenced by sex, age, skin type, presence of additional diseases, failure of previous treatment, or disease duration. The 308‐nm EL might be an alternative in the treatment of CHLP when the lesions are localized and in patients younger than 8 years old. The direct irradiation of 308‐nm EL can induce apoptosis of the T lymphocytes in LP skin lesions, thereby it has a unique therapeutic effect on the disease.^5^ Several cases of adult patients with oral lichen planus and lichen planopilaris treated effectively and safely with 308‐nm EL were reported.^5^, ^6^, ^7^, ^8^ In pediatric population, one patient with morphea‐lichen sclerosus and atrophicus overlap was treated with 308‐EL with a satisfactory response.^9^ However, to our knowledge there are no reported cases of CHLP treated with 308‐nm EL.

Few side effects have been reported with EL therapy and they are consistent with adverse reactions associated with other forms of phototherapy including erythema, blistering, hyperpigmentation, and hypopigmentation.^10^

In our case, we chose EL over other treatments for the following reasons: it is a pain‐free technique compared to intralesional triamcinolone where the injections might be traumatizing giving the young age of the patient. Unlike conventional UVB phototherapy, EL is a targeted treatment that has the advantage of allowing the exposure of involved areas only, thus minimizing acute side effects and long‐term risk of skin cancer on unaffected skin as well as higher doses of energy can be delivered selectively to the lesions thereby enhancing efficacy and achieving faster response.^11^ Concerning oral antihistamines, we did not find the need to use them because the lesions where not itchy and topical calcineurin inhibitors are still not available in our country.

In summary, our case of CHLP is distinguished by the absence of pruritus and the unilateral presentation of the lesions. Besides, our case is the first to suggest that the 308‐nm EL is an effective, safe, and practical tool in the treatment of CHLP especially in very young patients and localized lesions. Further studies including a large number of patients are necessary to provide further data about the efficacity and the safety of the EL in the treatment of CHLP. However, it is important to highlight that EL is still an expensive device with limited access especially in developing countries which makes it difficult to be chosen as a popular alternative treatment.

## CONFLICT OF INTEREST STATEMENT

The authors declare that there are no conflicts of interest in this work.

## ETHICS STATEMENT

The authors confirm that the ethical policies of the journal, as noted on the journal’s author guidelines page, have been adhered to. No ethical approval was required as this is a review article with no original research data.

## CONSENT

The examination of the patient was conducted according to the principles of the Declaration of Helsinki. The authors certify that they have obtained all appropriate patient consent forms, in which the patient gave his consent for images and other clinical information to be included in the journal. The patient understands that his name and initial will not be published and due effort will be made to conceal his identity, but that anonymity cannot be guaranteed.

## Data Availability

Data sharing is not applicable to this article as no new data were created or analyzed in this study.
